# Atypical Histiocytoid Sweet Syndrome in a Patient With Crohn's Disease

**DOI:** 10.14309/crj.0000000000001154

**Published:** 2023-10-03

**Authors:** Phi Tran, Nikhil Seth, Maleka Najmi, Martin Fernandez, Christopher Johnson

**Affiliations:** 1 Department of Medicine, Baylor Scott & White Medical Center, Temple, TX; 2 Department of Pathology, Baylor Scott & White Medical Center, Temple, TX; 3 Department of Gastroenterology, Department of Medicine, Baylor Scott & White Medical Center, Temple, TX

## CASE REPORT

**Figure 1.**
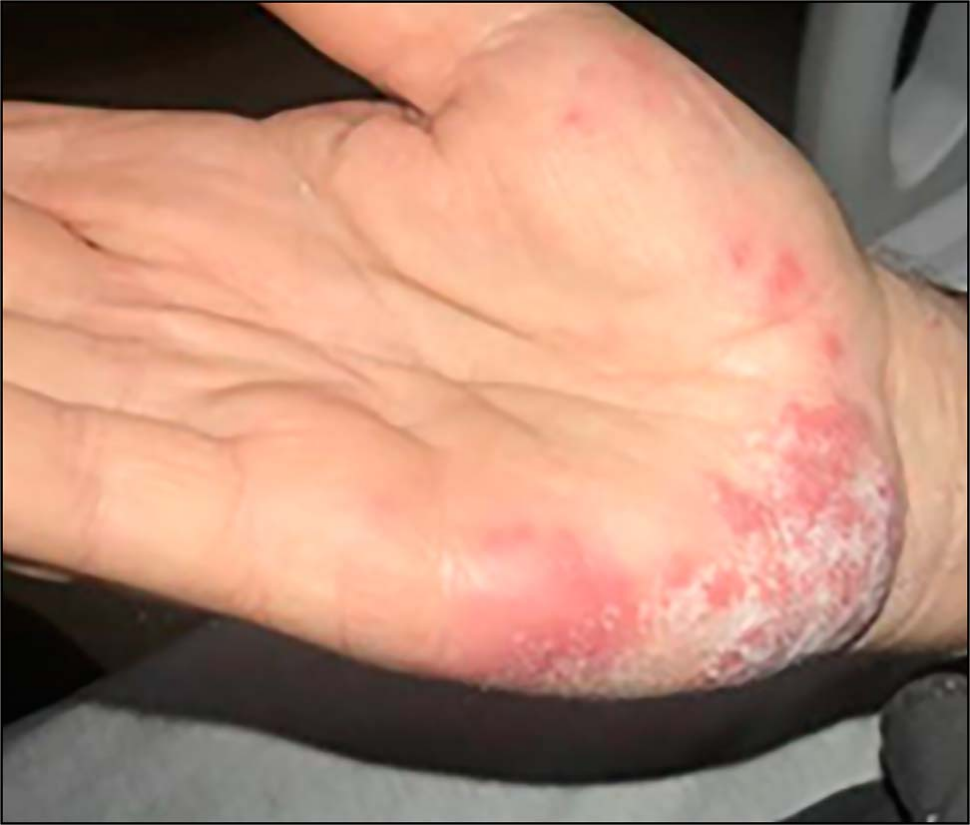
Violaceous tender plaques on the bilateral ulnar aspects of the palms.

**Figure 2.**
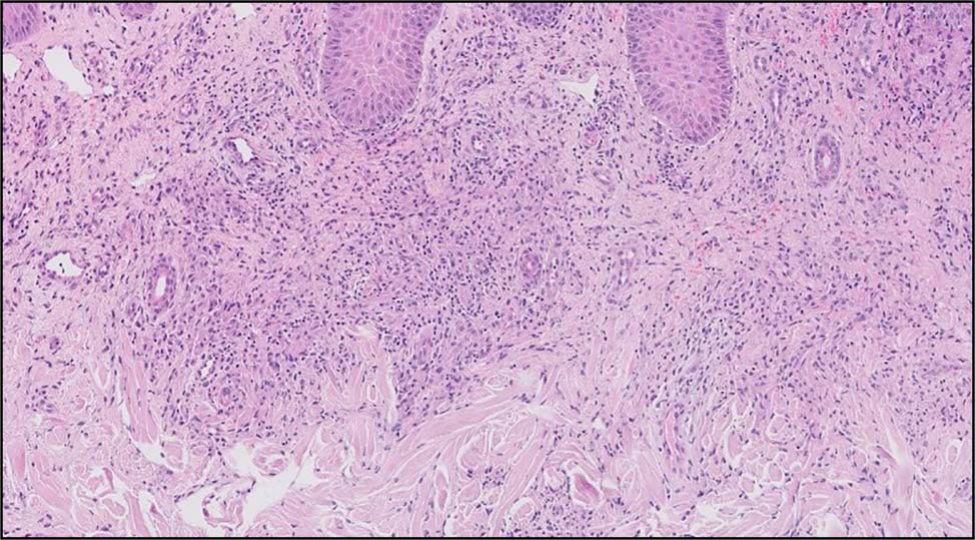
Punch biopsy of the right palm, revealing a superficial perivascular and interstitial infiltration composed of lymphocytes, histiocytes, and neutrophils with papillary dermal edema and red blood cell extravasation without signs of vasculitis (original magnification 400×).

A 65-year-old man with diabetes mellitus presented with hematochezia, a fever of 101.6°F, abdominal pain, and a new painful rash on his bilateral hands. Initial colonoscopy was suspicious for ulcerative colitis, but repeat colonoscopy revealed ulceration from the anal verge with skip lesions, making Crohn's disease more likely. Laboratory studies revealed a white blood cell count of 14.7 × 10^9^ g/L, C-reactive protein of 72.8 mg/L, and erythrocyte sedimentation rate of 92 mmol/h. Physical examination was remarkable for violaceous tender plaques on the bilateral ulnar aspects of the palms, third left proximal interphalangeal joint, right index pad, and left elbow (Figure 1). Punch biopsy of the right palm revealed a superficial perivascular and interstitial infiltration composed of lymphocytes, histiocytes, and neutrophils with papillary dermal edema and red blood cell extravasation without signs of vasculitis (Figure 2). Histiocytoid Sweet syndrome associated with Crohn's disease was diagnosed. Improvement of both Crohn's flare and his rash were seen after intravenous steroid administration. A month later, red patches were still present on the bilateral ulnar aspects of the palm that were now nontender. Sweet syndrome in Crohn's disease has been sparsely documented in the literature. Even more rare is the histiocytoid variant of Sweet syndrome in patients with Crohn's disease.

## DISCLOSURES

Author contributions: All authors listed above have made substantial contributions to the conception of the work, drafting the work or revising it critically for important intellectual content, final approval of the version to be published, and agreement to be accountable for all aspects of the work. P. Tran is the article guarantor.

Financial disclosure: None to report.

Informed consent was obtained for this case report.

